# A Composite Hydrogel with High Mechanical Strength, Fluorescence, and Degradable Behavior for Bone Tissue Engineering

**DOI:** 10.3390/polym11071112

**Published:** 2019-07-01

**Authors:** Yanqin Wang, Yanan Xue, Jinghui Wang, Yaping Zhu, Yu Zhu, Xuehui Zhang, Jingwen Liao, Xiaona Li, Xiaogang Wu, Yi-Xian Qin, Weiyi Chen

**Affiliations:** 1 College of biomedical engineering, Taiyuan University of Technology, Taiyuan 030024, China; 2 State Key Laboratory of Luminescent Materials and Devices, South China University of Technology, Guangzhou 510640, China; 3 State Key Laboratory of Molecular Engineering of Polymers, Fudan University, Shanghai 200433, China; 4 Sixth Academy of China Aerospace Science & Industry Corporation, Hohhot 010010, China; 5 Department of Biomedical Engineering, State University of New York at Stony Brook, New York, NY 11794, USA

**Keywords:** double-network, hydrogel, mechanical strength, fluorescence, degradable

## Abstract

In this work, to obtain a novel composite hydrogel with high mechanical strength, fluorescence and degradable behavior for bone tissue engineering, we prepare a nanofiller and double-network (DN) structure co-enhanced carbon dots/hydroxyapatite/poly (vinyl alcohol) (CDs/HA/PVA) DN hydrogel. The composite hydrogels are fabricated by a combination of two fabrication techniques including chemical copolymerization and freezing‒thawing cycles, and further characterized by FTIR, XRD, etc. Additional investigations focus on the mechanical properties of the hydrogel with varying mass ratios of CDs to PVA, HA to PVA and different numbers of freezing/thawing cycles. The results show that the as-prepared CDs_3.0_/HA_0.6_/PVA DN_9_ hydrogel has optimized compression properties (Compression strength = 3.462 MPa, Young’s modulus = 4.5 kPa). This is mainly caused by the synergism effect of the nanofiller and chemical and physical co-crosslinking. The water content and swelling ratio of the CDs/HA/PVA SN and DN gels are also systematically investigated to reveal the relationship of their microstructural features and mechanical behavior. In addition, in vitro degradation tests of the CDs/HA/PVA DN hydrogel show that the DN hydrogels have a prominent degradable behavior. So, they have potential to be used as high-strength, self-tracing bone substitutes in the biomedical engineering field.

## 1. Introduction

In the tissue engineering field, optimizing the mechanical properties and evaluating the degradation behavior of the bone substitute for orthopedic implants have been considered the most significant mission [1]. Hydrogels [2,3,4] have attracted more and more attention in the biomedical engineering field. However, it is urgent to improve their relatively poor mechanical properties for them to be applied as bone substitute materials [5,6,7]. Recently, several methods have been reported relating to the preparation of the PVA hydrogels, such as electron-beam irradiation [8], chemical cross-linking methods [9], and freezing/thawing cycles [10]. The chemical cross-linking method could fabricate PVA gels with higher thermal stability through covalent binding. However, the relatively high content of the crosslinker (such as glutaraldehyde) would inevitably lead to the gels being unsafe to use in living organisms. As for the cyclic freezing/thawing technique, it could form PVA crystal nuclei and hydrogen bonding, which could act as physical cross-linking points within PVA hydrogels without using any chemical crosslinkers. However, a PVA hydrogel prepared by this way would appear to have lower thermal stability. This would limit its further application in the tissue engineering field. In this work, to optimize biosafety and thermal stability and enhance the mechanical strength of the PVA hydrogel simultaneously, we combine chemical crosslinking (with a rather low ratio of crosslinker) with cyclic freezing/thawing technique for a chemical and physical co-crosslinked double-network (DN) hydrogel.

Moreover, due to the good biocompatibility and osteointegration [11], in this work, nano-hydroxyapatite (HA) has been chosen as a nanofiller to strengthen the mechanical properties of the PVA hydrogel. In addition, in the biomedical engineering field, the bone substitute has been endowed with desirable self-tracing properties, so the deformation and degradation behavior can be tracked more conveniently and intuitively [12,13]. The introduction of a nanofiller with fluorescent properties is a widely accepted technique that can not only improve the mechanical properties but also endow fluorescence signals to the hydrogels [14]. For example, in Zhi’s work [15], a Eu^3+^ organic complex was introduced into polyvinyl acetate (PVAc), which was a precursor polymer of PVA. After hydrolysis, the Eu-containing PVA was obtained, and its aqueous solution formed the hydrogels with boric acid. The as-prepared hydrogels had good strength and photoluminescence properties. However, considering that the Eu^3+^ that degraded from the hydrogel could be a potential hazard for living organisms, this composite hydrogel was not suitable to be used as a bone substitute in vivo. Carbon dots (CDs) [16] have relatively high fluorescence stability and resistance to photo degradation and photo bleaching, so they have been widely applied in bioimaging [17]. Recently, Hu’s group [18] prepared doped PVA/C-dot hydrogels by the physical crosslinking technique—cyclic freezing/thawing of the aqueous suspension of PVA and C-dots. However, this composite hydrogel existed only as a hydrogen bond-based polymer network; when used in the bone tissue engineering field, both the mechanical strength and osteoconductive ability were far from satisfactory.

In this work, we develop a novel method for the preparation of CDs/HA/PVA DN hydrogel with highly mechanical strength, degradable, and fluorescence emission properties. The CDs/HA/PVA DN hydrogel with different composite ratios are optimized so that they have suitable surface functional groups, good compressive strength and viscoelastic behavior, fluorescence-visible properties, and degradable behavior, which are beneficial to them being used as visualization bone substitutes in the biomedical engineering field.

## 2. Materials and Methods

### 2.1. Materials

PVA (DS = 0.61 and number-average polymerization degree of 1799, 98~99%), hydroxyapatite (HA) > 12 nm particle size and α-d-lactose were analytical grade and received from Aladdin Chemistry Co. (Shanghai, China). Tris (hydroxymethyl) amino-methane (Tris) (99.9%) and HCl were analytical grade reagents purchased from Solarbio (Beijing, China) and used as supplied. Glutaraldehyde was obtained from Aladdin Chemistry Co. (Shanghai, China). Deionized water was used in all experiments.

### 2.2. Methods

#### 2.2.1. Preparation

##### Preparation of the Carbon Dots (CDs)

The CDs were prepared as described previously [16]. Briefly, 0.1 g of lactose and 2.5 g of Tris-base were dissolved in 100 mL pure water. The pH of the mixed solution was adjusted to about 10.5 and it was hydrothermally refluxed at 100 °C under stirring for 24 h. The solution turned from colorless to yellow. After being cooled to room temperature, the CD solution was dialyzed (MW 1000) against water for two days. Finally, the pure C-dot power was obtained by freeze-drying the purified C-dot suspension.

##### Preparation of the CDs/HA/PVA Composite Hydrogel

A two-step method was used to synthesize the CDs/HA/PVA DN composite hydrogels. In brief, a PVA solution was prepared by dissolving 6.0 g of PVA powder in 100 mL distilled water at 90 °C. Then, a CD solutions (0–2 mL) and nano-hydroxyapatite (HA) powder (0.05–0.4 g) were added to the PVA solution, and the mixture was stirred under sonication to be homogeneous, with glutaraldehyde (0.1 mL), and HCl (10%, 500 μL) then added. The resulting solution was poured into a mold and placed in a water bath at 50 °C for 1 h to form chemically crosslinked CDs/HA/PVA single network (SN) hydrogels. Then the chemically crosslinked SN hydrogels experienced repeated (0–9 times) freezing/thawing cycles (freezing at −20 °C for 10 h; thawing at 25 °C for 14 h) to obtain chemically and physically co-crosslinked CDs/HA/PVA DN hydrogels.

#### 2.2.2. Fourier-Transform Infrared Spectroscopy (FTIR)

FTIR spectra of CDs, HA and CDs/HA/PVA composites were recorded in a Bruker VERTEX70 spectrometer (Karlsruhe, Germany) between 400 cm^−1^ and 4000 cm^−1^ at a resolution of 4 cm^−1^.

#### 2.2.3. X-Ray Diffraction (XRD) Measurement

The XRD of the CDs powder, CDs/PVA hydrogel and CDs/HA/PVA composite hydrogel were measured using an X-ray diffractometer (TD-3500, Dandong Tongda Science and Technology Co., Ltd.) (Dandong, China) with 45 kV voltage and 30 mA current. The samples were 2 mm thick films and measured from 5° to 50° (2θ) in steps of 0.05°.

#### 2.2.4. Swelling Behavior Characterization

Swelling behaviors were carried out in distilled water. The equilibrium swelling degree (W%) was determined by the following equation: W% = (M_2_ − M_1_)/M_1_ × 100%, where M_1_ is the weight of the dry samples before immersion and M_2_ is the weight of the sample after absorption of water at the equilibrium.

#### 2.2.5. Water Content of the Composite Hydrogel

To measure the water content of the CDs/HA/PVA hydrogel with different CD contents, the samples (column, diameter 10 mm and height 2 mm) were dried to a constant weight in a vacuum at 60 °C for 24 h. The water content was calculated as follows: Water content% = (W_0_ − W)/W_0_ × 100%, where W_0_ and W are the weight of the CDs/HA/PVA hydrogel before and after drying, respectively.

#### 2.2.6. Mechanical Property Measurements

The mechanical properties of the CDs/HA/PVA composite gels were tested using a Universal Testing Machine (Instron 3343, Boston, MA, USA). The tensile strength of the hydrogels (width 4 mm and length 30 mm) was measured at a strain rate of 30 mm min^−1^ until breaking occurred. For the compression tests, the hydrogel samples (cylindrical, diameter 12 mm and height 15 mm) were placed between the self-leveling plates and compressed at a rate of 10 mm min^−1^ until the compression ratio was 85%. The Young’s modulus was calculated from the slope of the initial linear region of the stress-strain curves (5–15% strain). For each sample, a minimum of three measurements was taken and the average value was reported. The nominal stress and strain, σ and ε, were recorded as the applied load divided by the original cross-sectional area of the samples and the clamp displacement divided by L_0_. For the hysteresis test, the loading and unloading cycles were performed at a constant compression rate (10 mm min^−1^) to maintain a unique loading history.

#### 2.2.7. Degradation Properties of the Hydrogel

Degradation behaviors of the CDs/HA/PVA DN composite gels with different numbers of freezing‒thawing cycles (0, 3, 5, 7, or 9) were measured. All the freeze-dried samples were weighed (W_0_) and soaked in bottles with 50 mL of fetal bovine serum solution and incubated at 37 °C for 5, 10, 15, 20, 30, and 40 days. Then all the samples were retrieved, rinsed with deionized water, frozen, freeze-dried, and weighed (W_t_). The weight loss percentage (W%) was calculated according to the formula W% = (W_0_ − W_t_)/W_0_ × 100%.

## 3. Results

### 3.1. Preparation and Properties of the Hydrogels

**Scheme 1:**
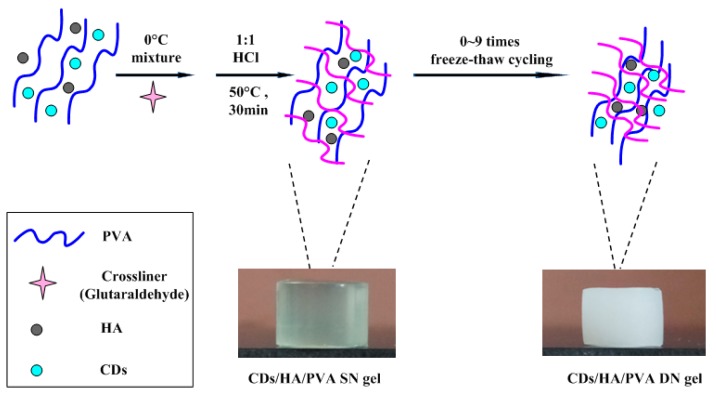
The design strategy for the CDs/HA/PVA DN hydrogel.

The CDs/HA/PVA double-network (DN) hydrogels were fabricated in two stages, including chemical copolymerization and cyclic freezing/thawing. In the first stage, with the presence of HA and CDs as nanofillers, PVA chains covalently crosslinked at a rather low concentration (considering the biosafety) of glutaraldehyde for the first chemical-crosslinked network, the as-prepared gels were defined as a CDs/HA/PVA single network (SN) gel. In the second stage, during the cyclic freezing‒thawing process, the PVA crystalline domains serve as cross-linking knots and hydrogen bonds were formed between the PVA chains for the second physically crosslinked network; then the gel was defined as the CDs/HA/PVA double-network (DN) gel. The design strategy is shown in Scheme 1. In this paper, all hydrogels are expressed as CDs_X_/HA_Y_/PVA DN_n_ gels, where X[%] refers to the mass ratio of CDs relative to PVA, Y refers to the mass ratio of HA relative to PVA, and *n* refers to the number of freezing/thawing cycles.

**Figure 1:**
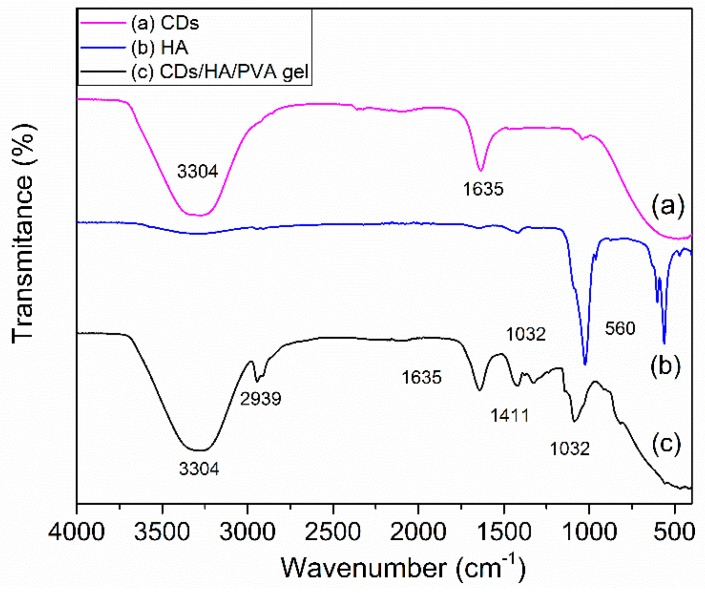
The FTIR spectra of dried (**a**) CDs powder, (**b**) HA powder, and (**c**) CDs/HA/PVA hydrogel.

To confirm the interaction between the CDs, HA, and PVA chains, FTIR spectra of the dried CDs, HA, and CDs/HA/PVA composite gels were investigated as shown in Figure 1. The CDs (curve a) exhibited characteristic absorption bands of O–H stretching vibrations at 3304 cm^−1^, and amide bond vibrations at 1635 cm^−1^. The HA (curve b) exhibited characteristic absorption bonds of C‒O‒C in the carboxylate groups at about 1032 cm^−1^. In addition, in curve c, the CDs/HA/PVA gel exhibited characteristic absorption bonds O‒H stretching vibrations at 3304 cm^−1^, C‒H stretching vibrations at 2939 cm^−1^, amide bond vibrations at 1631 cm^−1^, and C‒O‒C stretching vibrations at about 1411 cm^−1^ and 1032 cm^−1^ [19]. The characteristic peaks derived from CDs, HA, and PVA have all appeared, which meant the successful formation of hybrid CDs/HA/PVA hydrogels. Due to the CDs being prepared using α-d-lactose as the carbon source, there were plenty of ‒OH groups on their surfaces, which tended to interact with the ‒OH of the PVA networks, so the CDs could be firmly entrapped inside the PVA networks through hydrogen-bonding interactions [20,21]. The hydrogen bonding between the hydroxyl groups of HA and PVA chain could also entrap the HA within the PVA networks tightly.

**Figure 2:**
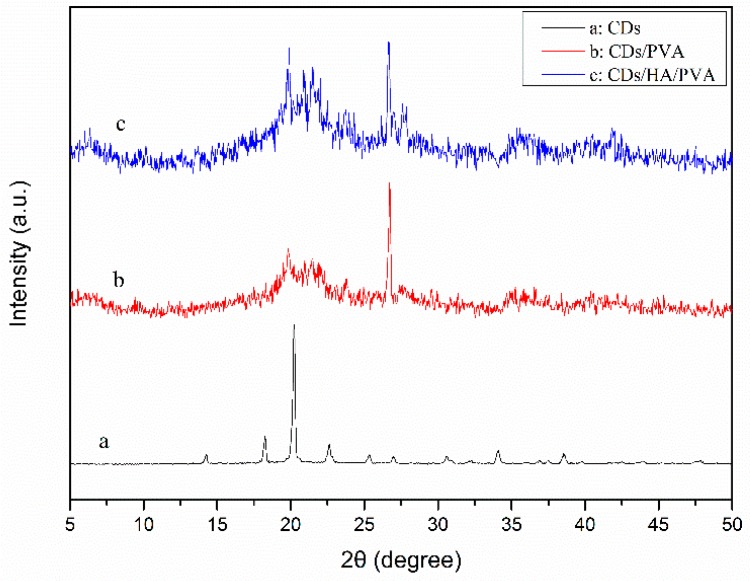
The XRD patterns of dried (**a**) CDs, (**b**) CDs/PVA hydrogel, and (**c**) CDs/HA/PVA hydrogel.

The X-ray diffraction patterns of the carbon dots (CDs), CDs/PVA, and CDs/HA/PVA hydrogels were also investigated for the effect of CDs and HA on the crystallinity degree of PVA hydrogel. As shown in Figure 2, a prominent peak was seen at 2θ = 21.7° for CDs (curve a) [22]. The crystallization peak of PVA at 19.6° was not obvious, while the peak of HA at 26° was rather apparent for the CDs/PVA hydrogel (curve b). For the CDs/HA/PVA hydrogels (curve c), there were three characteristic peaks at 19.4°, 21.7°, and 26°, which meant there were crystalline HA and CDs within the semi-crystalline PVA hydrogel.

### 3.2. Mechanical Properties of the Hydrogels

#### 3.2.1. Mechanical Properties of the Hydrogels with Different Mass Ratios of CDs to PVA

**Figure 3:**
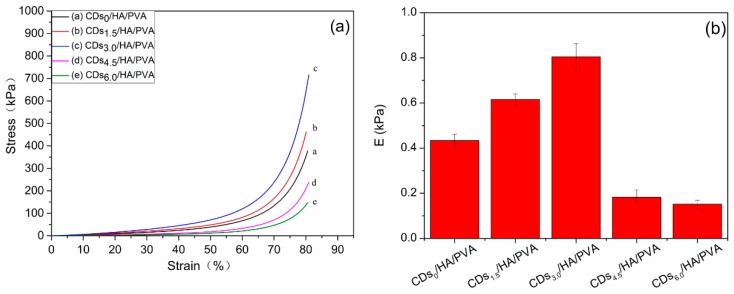
(**a**) The compression stress‒strain curves and (**b**) Young’s modulus of the CDs/HA_0.6_/PVA SN hydrogels with different mass ratios of CDs to PVA at (**a**) 0.0%, (**b**) 1.5%, (**c**) 3.0%, (**d**) 4.5%, and (**e**) 6.0%, respectively.

For a composite hydrogel applied as a cartilage/bone substitute, the compressive mechanical property is an important parameter. Figure 3a shows the compression stress‒strain curve of the different mass ratios of the CDs to PVA. When the mass ratio of CDs to PVA was 3.0 wt %, the compression strength of the CDs/HA_0.6_/PVA SN hydrogels increased by about 81% at a strain of 80% compared with the HA/PVA gel without CDs. Its Young’s modulus increased by 90.2% compared with the HA/PVA gel without CDs (Figure 3b). However, the higher mass ratio of CDs to PVA, such as 4.5 wt % or 6.0 wt %, was harmful to the compression strength and Young’s modulus of the CDs/HA/PVA SN hydrogels. The possible mechanism for the phenomenon was that, with the increase in the CDs content, the cross-linking density of the CDs/HA/PVA SN hydrogel was greatly enhanced compared with the HA/PVA hydrogel due to strong hydrogen bonding interactions between the surface functional groups (‒OH) of the PVA and CDs, which led to an enhancement of the compression mechanical properties. However, the higher content of CDs leads to a denser and less homogeneous cross-linked network in the PVA hydrogel, which in turn decreases the mechanical properties of the HA/PVA hydrogel, as could also be seen in a previously published work [23].

**Figure 4:**
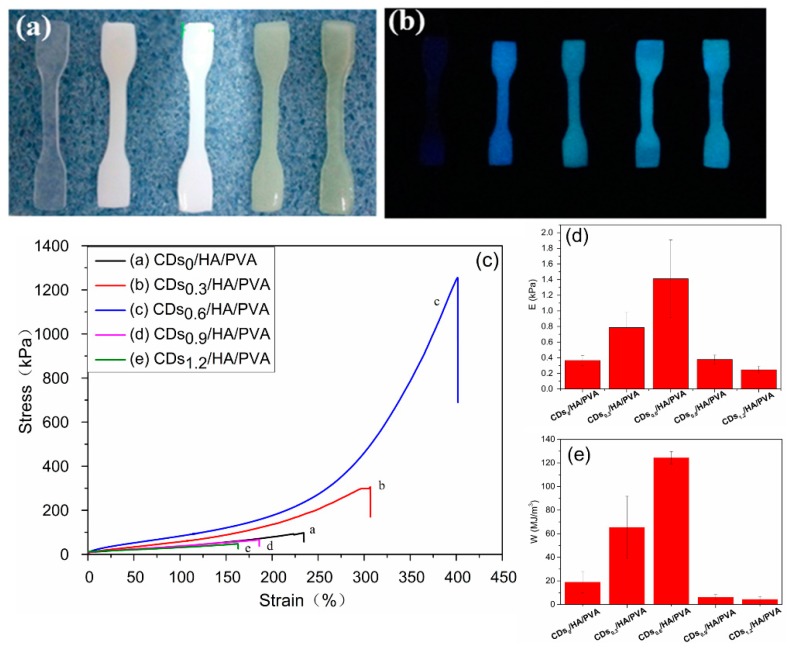
The tensile test-piece of the CDs/HA/PVA SN hydrogels with increasing mass ratios of CDs/PVA (0.0%, 0.3%, 0.6%, 0.9%, and 1.2%, respectively, from left to right (**a**) in the sunlight and (**b**) under UV light. (**c**) The tensile stress‒strain curves, (**d**) Young’s modulus, and (**e**) fracture energy of the CDs/HA_0.6_/PVA SN hydrogels with different mass ratios of CDs to PVA at 0.0%, 0.3%, 0.6%, 0.9%, and 1.2%, respectively.

Moreover, the appearance of CDs could also endow hydrogel with excellent fluorescence properties. As show in Figure 4b, the tensile test of all the CDs/HA/PVA SN hydrogel kept a smooth 3D shape and obvious blue fluorescence emission under UV light (λ = 300 nm). The fluorescence spectra of the CDs and the CDs/HA/PVA hydrogels with different CD contents are shown in Figure S1. In addition, the tensile strength of the CDs/HA_0.6_/PVA SN hydrogel remarkably increased at first and then decreased with the increasing mass ratios of CDs to PVA (as shown in Figure 4c). The mass ratio of CDs to PVA at 0.6 wt% could effectively improve the mechanical properties of the PVA hydrogel; about an 1150% increase in tensile strength and a 76.1% increase of elongation at break were achieved compared to the HA/PVA gel without CDs (curve a). In addition, the Young’s modulus and fracture energy of the CDs/HA/PVA SN hydrogels were about 1.4 kPa and 124.4 MJ/m^3^, increases of nearly 300% and 555.4%, respectively. Further increments led to a mass ratio of CDs to PVA of 0.9 wt % and 1.2 wt %, while the tensile strength, Young’s modulus, and fracture energy of the CDs/HA/PVA SN hydrogels appeared to decrease even lower than the HA/PVA gel without CDs.

#### 3.2.2. Mechanical Properties of Hydrogels with Different Mass Ratios of HA to PVA

**Figure 5:**
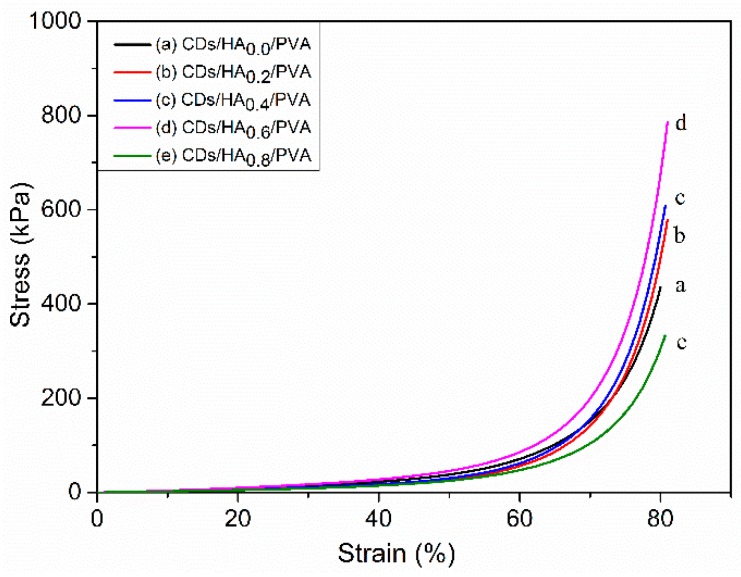
The compression stress‒strain curves of the CDs_3.0_/HA/PVA SN hydrogels with different mass ratios of HA to PVA at (**a**) 0.0, (**b**) 0.2, (**c**) 0.4, (**d**) 0.6, and (**e**) 0.8, respectively.

The impact of the HA on the mechanical properties of the CDs/HA/PVA hydrogels was also investigated as shown in Figure 5. This shows the compression stress‒strain curves of the CDs_3.0_/HA/PVA SN hydrogels with different mass ratios of HA to PVA. The compression strength was enhanced as the mass ratio of HA to PVA increased to 0.6% and then decreased with further HA addition. This was due to the addition of HA modifying the physical and chemical features of the hydrogel and promoting material cross-linking and stability. The agglomeration of HA would obviously reduce the specific surface area, which would reduce the interfacial bonding strength between the CDs and the PVA macromolecules, and ultimately result in a deterioration of the mechanical properties of the CDs/HA/PVA composite hydrogels.

#### 3.2.3. Mechanical Properties of the Hydrogels with Different Numbers of Freezing/Thawing Cycles

**Figure 6:**
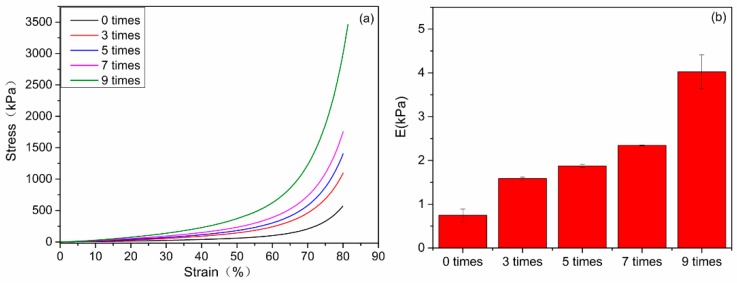
(**a**) The compression stress‒strain curves and (**b**) Young’s modulus of the CDs_3.0_/HA_0.6_/PVA DN hydrogels with 0, 3, 5, 7, and 9 freezing/thawing cycles, respectively.

The CDs/HA/PVA DN hydrogels were further fabricated by 3–9 freezing/thawing cycles of the as-prepared CDs_3.0_/HA_0.6_/PVA SN hydrogels. Compared with the CDs_3.0_/HA_0.6_/PVA SN hydrogel without freezing/thawing, the increase in the number of freezing/thawing cycles led to a prominent enhancement of the compression strength (Figure 6). After nine freezing–thawing cycles, the optimal mechanical properties of the CDs/HA/PVA DN hydrogels were obtained: the compression stress was at 3462 kPa, and the Young’s modulus was at 4.5 kPa (Figure 6b). Compared with the CDs/HA/PVA gels without freezing/thawing, they exhibited about a 477% increase in compression strength at a strain of 80% and a 429% increase in the Young’s modulus. The possible mechanism was that the freezing‒thawing process could form more PVA crystal nuclei, which acted as physical cross-linking points in PVA hydrogels, thereby increasing the cross-linking degree of the DN gel. More information about the feed compositions as well as the major parameters of the fabricated CDs/HA/PVA gel samples with varying mass ratios of CDs to PVA and HA to PVA are summarized in Table S1.

**Figure 7:**
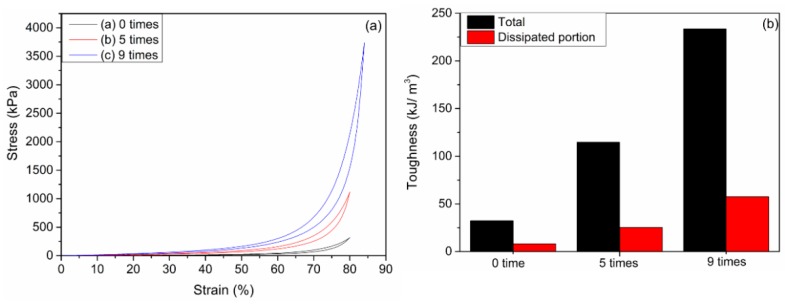
(**a**) Loading‒unloading stress‒strain curves and (**b**) the total toughness and dissipated toughness of the CDs_3.0_/HA_0.6_/PVA DN hydrogel after 0, 5, and 9 freezing‒thawing cycles at a strain of 80%. The compression velocity was fixed at 10 mm min^−1^.

In addition, the loading-unloading tests were measured to evaluate the energy dissipation of the CDs_3.0_/HA_0.6_/PVA DN hydrogels. The area between the loading and unloading curves of a hydrogel gives the energy dissipated per unit volume. Figure 7a shows the compression loading‒unloading curves and (b) the total toughness and dissipated toughness of the CDs_3.0_/HA_0.6_/PVA DN hydrogels after 0, 5, and 9 freezing‒thawing cycles. Obvious hysteresis loops were observed for the hydrogels with five cycles, but the hydrogel with nine freezing‒thawing cycles has a much larger hysteresis loop, suggesting it has a much more effective energy dissipation pathway.

### 3.3. The Water Content and Swelling Behavior of the SN and DN Hydrogels

**Figure 8:**
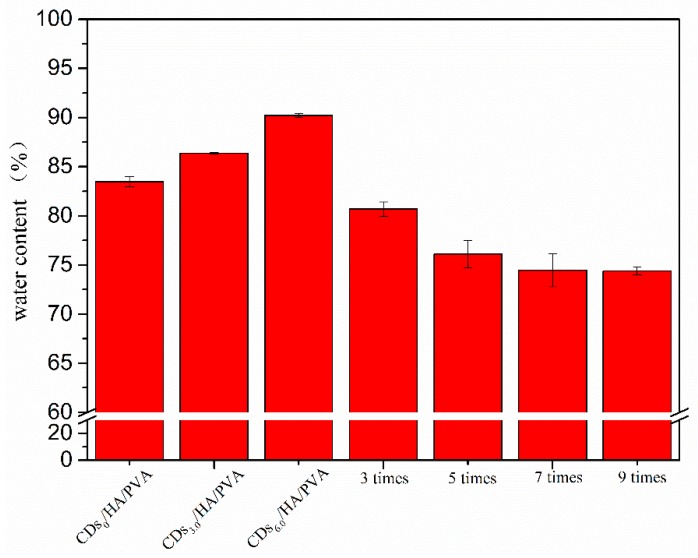
The water contents of the CDs/HA_0.6_/PVA SN hydrogels with different mass ratios of CDs to PVA at 0%, 3.0%, and 6.0% and CDs_3.0_/HA_0.6_/PVA DN hydrogels with 3, 5, 7, and 9 freezing/thawing cycles, respectively.

The water content was essential to the mechanical properties of hydrogels. Figure 8 shows the water content of the CDs/HA_0.6_/PVA SN hydrogel with different percentages of CDs to PVA (0.0%, 3.0%, and 6.0%) and the CDs_3.0_/HA_0.6_/PVA DN hydrogel with different numbers of freezing/thawing cycles (0, 3, 5, 7, and 9). From Figure 8, we can see that all the CDs/HA/PVA SN hydrogels with increasing mass ratios of CDs to PVA and freezing/thawing cycles showed a high water content (above 70%). With the mass ratios of CDs to PVA increasing from 0% to 6.0%, the CDs/HA/PVA SN hydrogels showed increasing water content, from 83% to 90%. For the CDs_3.0_/HA_0.6_/PVA DN gels with an increased number of freezing/thawing cycles (from 0 to 9), their water content showed a prominent decrease from 90% to 74%. This was due to both the CDs content and freezing‒thawing cycle times that could influence the crosslinking degree of the PVA network [24] and further affect the water content.

**Figure 9:**
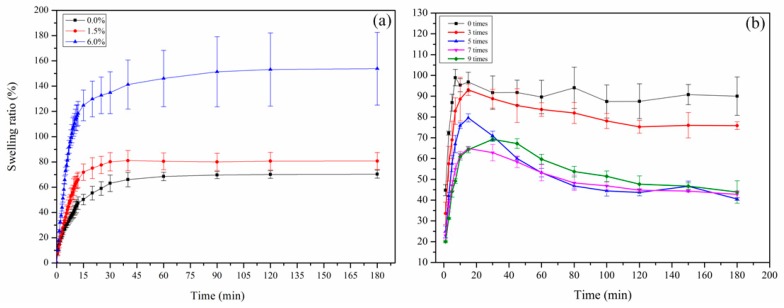
The swelling ratios of (**a**) the CDs/HA/PVA DN hydrogels with mass ratios of CDs to PVA at 0%, 1.5%, and 6.0% and (**b**) CDs_3.0_/HA_0.6_/PVA DN hydrogels with 0, 3, 5, 7, and 9freezing/thawing cycles, respectively.

The swelling behaviors of the CDs/HA/PVA SN and DN were also systematically investigated to reveal the difference of their microstructural features. As can be seen in Figure 9a, for the CDs/HA/PVA SN hydrogels with different mass ratios of CDs to PVA of 0%, 1.5%, and 6.0%, they all achieved swelling equilibrium within 60 min. The SN gels with higher CDs content reached a higher swelling ratio at the equilibrium stage. For example, the swelling ratios of the CDs_6.0_/HA_0.6_/PVA SN hydrogels increased quickly within the initial 15 min and remained stable at nearly 150% when they reached equilibrium. It is well known that the swelling behavior of the hydrogel depends on the free hydroxyl groups (‒OH) and crosslink density of the gel network [25]. The CDs are having plenty of ‒OH groups could increase the hydrophilic properties of the gel and the swelling ratio. Similarly, CDs/HA/PVA DN hydrogels with more freezing/thawing cycles (from 0 to 9) have a higher crosslinking density, which was a disadvantage for the water absorption, so they also appeared to undergo a decrease in swelling ratios (as shown in Figure 9b).

### 3.4. Degradation Behavior of the CDs/HA/PVA DN Hydrogels with Different Numbers of Freezing/Thawing Cycles

**Figure 10:**
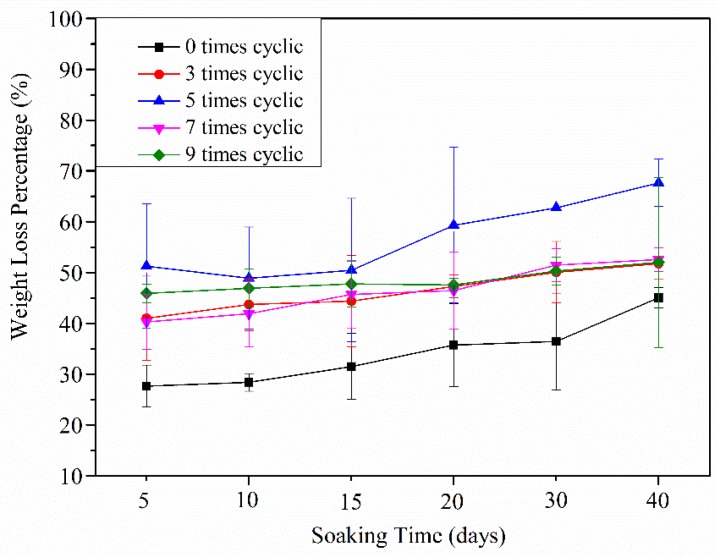
The degradation rate of the CDs_3.0_/HA_0.6_/PVA DN hydrogels with 0, 3, 5, 7, and 9 freezing‒thawing cycles after soaking in the fetal calf serum solution for 5, 10, 15, 20, 30, or 40 days.

Degradation of the polymeric biomaterials was also a critical factor in tissue engineering applications. In vitro degradation testing of CDs_3.0_/HA_0.6_/PVA DN hydrogel with different numbers of freezing/thawing cycles was carried out at 37 °C in a fetal bovine serum solution. All samples were dried and weighed to determine their weight loss percentages. As shown in Figure 10, the degradation trend of all the gels with different numbers of freezing/thawing cycles increased with soaking time from 5 to 40 days. The degradation that happened within the first five days was mainly due to the dissolving of the uncrosslinked PVA molecules, HA, and CDs particles. In addition, compared with the hydrogel groups with 0 or three freezing/thawing cycles, the DN gels with five freezing‒thawing cycles appeared to h have a higher degradation rate after the same number of soaking days. This was attributed to the higher cross-linking density and compact porous structures, which enabled the fetal bovine serum solution to penetrate easily into the hydrogel networks and further increased the degradation process. However, the degradation rates of the hydrogel groups with seven and nine freezing/thawing cycles showed a prominent decrease. This may be due to the gels with an exorbitant crosslinking degree not only limiting the rate and amount of solution penetration, but also slowing down the degradation process [26]. This indicated that the number of freezing/thawing cycles played an important role in the crosslinking degree of the DN gel, which further determined the degradation behavior of the DN gel.

## 4. Conclusions

In summary, CDs/HA/PVA DN hydrogels were successfully fabricated by combining chemical and physical co-crosslinking methods. FTIR and XRD characterization results confirmed the successful formation of hybrid CDs/HA/PVA hydrogels. After optimizing the mass ratios of CDs to PVA and HA to PVA, and the number of freezing/thawing cycles, we obtained CDs_3.0_/HA_0.6_/PVA DN_9_ hydrogels with the optimized compression mechanical properties (Compression strength = 3.462 MPa, Young’s modulus = 4.5 kPa), excellent fluorescence properties, and prominent degradation behaviors. All the CDs/HA/PVA SN hydrogels with increasing mass ratios of CDs to PVA and freezing/thawing cycles showed high water content (above 70%). The CDs/HA/PVA DN hydrogels with more freezing/thawing cycles (from 0 to 9) had higher crosslinking density and decreased swelling ratios. The CDs/HA/PVA DN hydrogels have the potential to be used as visualization bone substitutes in the biomedical engineering field.

## Supplementary Materials

The following are available online at https://www.mdpi.com/2073-4360/11/7/1112/s1. Table S1. The mechanical properties of the CDs/HA/PVA SN hydrogels with various proportions. Figure S1. (a) the fluorescence excitation and emission spectroscopy of the as-prepared CDs and (b) fluorescence emission spectroscopy of the CDs/HA_0.6_/PVA SN hydrogels with mass ratios of CDs to PVA at 0.0%, 0.6%, 0.9% and 1.2%, respectively (λ_ex_ = 300 nm).
