# Supplementary material for: A Composite Hydrogel with High Mechanical Strength, Fluorescence, and Degradable Behavior for Bone Tissue Engineering
Source: Polymers (Basel). 2019 Jul 1;11(7):1112. doi: 10.3390/polym11071112 (PMC6680580; doi:10.3390/polym11071112)
Supplement: Supplementary file 1 [file polymers-11-01112-s001.pdf]

Supporting information

A Composite Hydrogel with High Mechanical Strength, Fluorescence, and Degradable Behavior for Bone Tissue Engineering

Yanqin Wang [1,2,3]*, Yanan Xue [1,4], Jinghui Wang [1], Yaping Zhu [1], Yu Zhu [1], Xuehui Zhang [1], Jingwen Liao [1], Xiaona Li [1], Xiaogang Wu [1], Yi-Xian Qin [5] and Weiyi Chen [1]*

1 College of biomedical engineering, Taiyuan University of Technology, Taiyuan 030024, China; xueyanan5200@163.com (Y.X.); wangjinghui0783@link.tyut.edu.cn (J.W.); zhuyaping0928@link.tyut.edu.cn (Y.Z.); zhuyu0781@link.tyut.edu.cn (Y.Z.); zhangxuehui6983@link.tyut.edu.cn (X.Z.); ljwtyut@126.com (J.L.); lixiaona@tyut.edu.cn (X.L.); wuxiaogang@tyut.edu.cn (X.W.); yi-xian.qin@stonybrook.edu (Y.Q.)
2 State Key Laboratory of Luminescent Materials and Devices, South China University of Technology, Guangzhou 510640, China
3 State Key Laboratory of Molecular Engineering of Polymers, Fudan University, Shanghai 200433, China
4 Sixth Academy of China Aerospace Science & Industry Corporation, Hohhot 010010, China
5 Department of Biomedical Engineering, State University of New York at Stony Brook, New York, NY 11794, USA
* Correspondence: wangyanqin@tyut.edu.cn (Y.W.); chenweiyi@tyut.edu.cn (W.C.); Tel.: +86-351-3176655 (Y.W.); Fax: +86-22-23502458 (Y.W.)

Table 1. The mechanical properties of the CDs/HA/PVA SN hydrogels with various proportions.

Samples	Mass ratios of CDs to PVA (wt%)	Mass ratios of HA to PVA	Young's modulus (kPa)	Compression strength at a maximum strain of 85% (kPa)
$CDs_{0.0}/HA_{0.6}/PVA$	0.0	0.6	0.43±0.028	390.0±101.56
$CDs_{1.5}/HA_{0.6}/PVA$	1.5	0.6	0.62±0.025	470.2±36.26
$CDs_{3.0}/HA_{0.6}/PVA$	3.0	0.6	0.81±0.058	710.6±74.74
$CDs_{4.5}/HA_{0.6}/PVA$	4.5	0.6	0.18±0.031	220.7±102.31
$CDs_{6.0}/HA_{0.6}/PVA$	6.0	0.6	0.15±0.018	150.5±52.38
$CDs_{3.0}/HA_{0.0}/PVA$	3.0	0.0	0.43±0.043	435.7±8.13
$CDs_{3.0}/HA_{0.2}/PVA$	3.0	0.2	0.52±0.038	570.8±162.44
$CDs_{3.0}/HA_{0.4}/PVA$	3.0	0.4	0.32±0.006	610.7±44.60
$CDs_{3.0}/HA_{0.6}/PVA$	3.0	0.6	0.26±0.016	409.4±239.70
$CDs_{3.0}/HA_{0.8}/PVA$	3.0	0.8	0.25±0.017	330.6±64.54

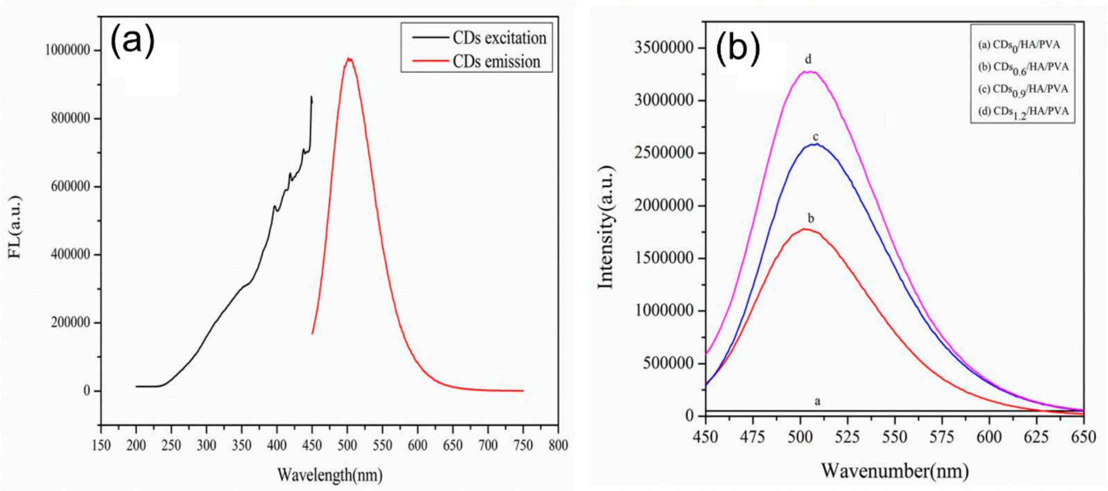

Figure S1. (a) the fluorescence excitation and emission spectroscopy of the as-prepared CDs and (b) fluorescence emission spectroscopy of the CDs/$HA_{0.6}$/PVA SN hydrogels with mass ratios of CDs to PVA at 0.0%, 0.6%, 0.9% and 1.2%, respectively (λex=300 nm).
